# Clinical Usefulness and Prognostic Value of Red Cell Distribution Width in Colorectal Cancer

**DOI:** 10.1155/2018/9858943

**Published:** 2018-12-13

**Authors:** Yanfang Song, Zhengyuan Huang, Yanli Kang, Zhen Lin, Pingxia Lu, Qing Lin, Zhaolian Cai, Yingping Cao, Xianjin Zhu

**Affiliations:** ^1^Clinical Laboratory, Department of Laboratory Medicine, The Affiliated People's Hospital of Fujian University of Traditional Chinese Medicine, 602 Bayiqi Road, Fuzhou 350001, China; ^2^Department of Emergency Surgery, Fujian Medical University Union Hospital, 29 Xinquan Road, Fuzhou 350001, China; ^3^Department of Laboratory Medicine, Fujian Medical University Union Hospital, 29 Xinquan Road, Fuzhou 350001, China

## Abstract

Red blood cell distribution width (RDW) indicates the heterogeneity in the size of circulating red blood cells. Increasing studies showed that RDW may be a diagnostic and prognostic marker in various tumors. To investigate the value of RDW as a biomarker in the diagnosis and prognosis of colorectal cancer (CRC), we evaluated 783 newly diagnosed CRC patients, 463 colorectal adenomas (CA) patients, and 331 healthy controls from June 2015 to October 2017 at Fujian Medical University Union Hospital. We found that RDW levels were significantly higher in CRC groups compared with both the CA and healthy control groups (*P<0.001*). Receiver-operating characteristic (ROC) analysis showed that the area under the ROC curve (AUC) for RDW, CEA, and CA19-9 was 0.643, 0.742, and 0.629 in discriminating CRC patients from healthy controls, respectively. When RDW cut-off value of 13.95 was applied, we distinguished CRC patients from healthy controls with a sensitivity of 41% and a specificity of 94%. Moreover, combined detection of RDW, CEA, and CA19-9 appeared to be a better diagnostic performance with a sensitivity of 56% and a specificity of 99%. However, RDW had little diagnostic value in the differential diagnosis between CRC patients and CA patients. More importantly, RDW levels were significantly associated with TNM stage, pT stage, pM stage, and tumor size among CRC patients. Overall, our study suggested that RDW might be an auxiliary biomarker for diagnosis and prognosis of CRC.

## 1. Introduction

Colorectal cancer (CRC) is the third most common cancer and the fourth leading cause of cancer-related deaths for Chinese people [1]. As a result of the aging population and unhealthy lifestyle, CRC is a growing public health problem in China. Although the underlying mechanisms of CRC are not fully understood, mortality from CRC can be reduced with appropriate screening. A number of CRC screening means, such as the fecal occult blood test, stool DNA test, colonoscopy, and computed tomography (CT) test, have been applied for years, but these screening means have lots of limitations, such as low sensitivity, low specificity, and being invasive or of high expense, which restrict their clinical application [2, 3]. Therefore, it is urgently needed to develop simple, inexpensive, and readily available biomarkers for the diagnosis and prognosis of CRC.

Red blood cell distribution width (RDW) is one of the routine laboratory parameters reported in the complete blood count test, and it indicates the heterogeneity in the size of circulating red blood cells [4]. Under normal conditions, the diameter of red blood cells is about 6-8 *μ*m; however, in some pathological cases, the diameter of red blood cells will have a variation. Higher RDW values indicate greater variation in the size of red blood cells. For several decades, RDW was easy to obtain with minimally invasive and widely used to make a differential diagnosis of anemia in clinical practices [5]. Recently, increasing studies showed that RDW may be a prognostic marker in various tumors [4], such as renal cell carcinoma [6], gastric cancer [7], lung cancer [8, 9], ovarian cancers [10], esophageal cancer [11], endometrial cancer [12], and breast cancer [13]. Besides, several studies have reported that RDW can be considered as a potential diagnostic marker in distinguishing malignant from benign tumors [14, 15].

To our knowledge, few reports have focused on the value of RDW in the diagnosis and prognosis of CRC. In 2004, Spell et al. firstly reported that RDW was elevated in 50% of colon cancer patients and elevated RDW may help better identify those patients who should be referred for full colonoscopy [15]. In addition, Ay et al. found that the level of RDW is significantly higher in colon cancer patients than in colon polyp patients, and RDW can be used as an early warning biomarker for solid colon tumors [16]. Moreover, two recent studies revealed that RDW is associated with cancer stage and survival in CRC patients [17–19]. These results suggested that RDW may be useful in predicting prognosis and also help the screening for CRC. However, the patient sample sizes of these studies were all less than 150, and small sample size makes it impossible to fully understand the value of RDW in CRC, indicating that the value of RDW in the diagnosis and prognosis of CRC is needed for further investigation in larger sample sizes.

In this study, we measured the level of RDW in 783 patients with CRC to analyze the value of RDW in the diagnosis of CRC. Furthermore, we evaluated whether RDW may have a potential role as a prognostic biomarker by evaluating the association between RDW and clinicopathologic characteristics in patients with CRC. Our study suggested that RDW has a potential role as a biomarker for the diagnosis and prognosis of CRC.

## 2. Materials and Methods

### 2.1. Patients' Characteristics

Medical records from 783 newly diagnosed CRC patients [467 men and 316 women, median age (interquartile range, IQR): 62 (54-68) years] were retrospectively reviewed in this study. All patients with CRC were diagnosed at Fujian Medical University Union Hospital (Fuzhou, China) from June 2015 to November 2017. Patients with anemia, hematologic disorder, and blood transfusion made in the last three months, receiving iron deficiency treatment and active inflammation, were excluded. 463 colorectal adenomas (CA) patients [312 men and 151 women, median age (IQR): 60 (51-67) years] and 331 healthy participants [162 men and 169 women, median age (IQR): 60 (50-68) years] were included as controls. This study was performed in accordance with ethical guidelines and was approved by the Institutional Medical Ethics Review Board of Fujian Medical University Union Hospital. Informed consent was obtained from all included participants.

### 2.2. Analysis of RDW, CEA, and CA19-9 Levels

Peripheral blood samples were obtained from newly diagnosed patients with CRC. RDW was measured routinely with Beckman Coulter LH 780 hematology analyzer (Beckman Coulter, Brea, CA, USA) according to the manufacturer's instructions. Serum levels of CEA and CA19-9 were measured using a Cobas 6000 Analyzer (Roche Diagnostics, Basel, Switzerland). According to the manufacturer's instructions, the cut-off value for normal CEA is less than 5 ng/mL, and that for normal CA19-9 is less than 37 U/mL.

### 2.3. Statistical Analysis

Statistical analysis was performed using SPSS version 21.0 software or GraphPad Prism version 5.0. The distribution of the RDW values is abnormal and RDW values are summarized as median with interquartile range (IQR). Between-group differences were tested using the Mann-Whitney U test. The diagnostic value of RDW was estimated by receiver-operating characteristic (ROC) curves. The Youden index was used to determine the optimal cut-off value for RDW to differentiate between CRC patients and healthy controls or CA patients. The relationship between RDW and clinicopathological characteristics was assessed by chi-squared test. All tests were two-tailed and a* p* value less than 0.05 was considered statistically significant.

## 3. Results

### 3.1. The Level of RDW in CRC Patients

**Figure 1:**
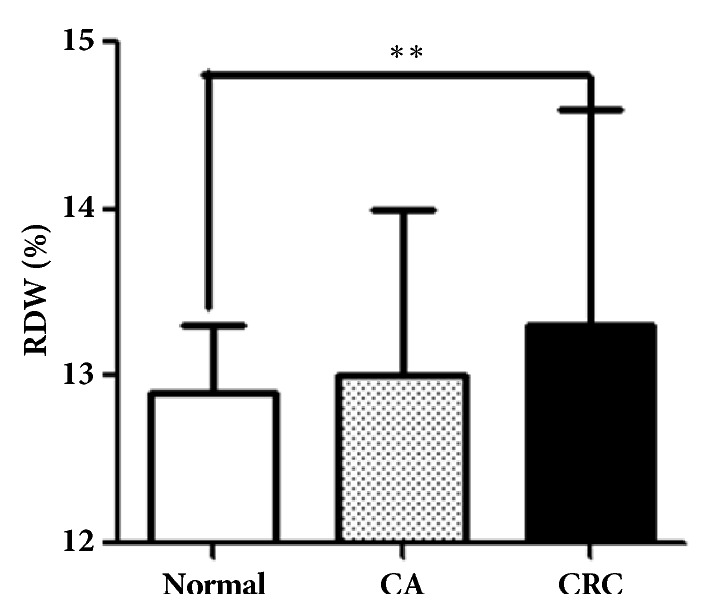
The level of RDW in CRC patients. RDW levels were determined by hematology analyzer in CRC patients (n= 783), colorectal adenoma patients (n = 463), and healthy controls (n = 331). Data are presented as median with interquartile range. ^∗∗^*p< 0.01*.

As shown in Figure 1, the median RDW were 13.3 (IQR: 12.7 - 14.6), 13.0 (IQR: 13.0 - 14.0), and 12.9 (IQR: 12.6 - 13.3) in CRC patients, CA patients, and healthy participants, respectively. The level of RDW was significantly higher in CRC patients than in healthy participants (CRC vs. healthy participants,* P < 0.001*). However, no significant difference was observed between CRC and CA patients.

### 3.2. Evaluation of RDW as a Potential Diagnostic Biomarker for CRC

We evaluated the value of RDW as a biomarker for the clinical diagnosis of CRC compared with carcinoembryonic antigen (CEA) and carbohydrate antigen 19-9 (CA19-9), which are the most commonly used serum tumor markers in the diagnosis of CRC.

**Figure 2:**
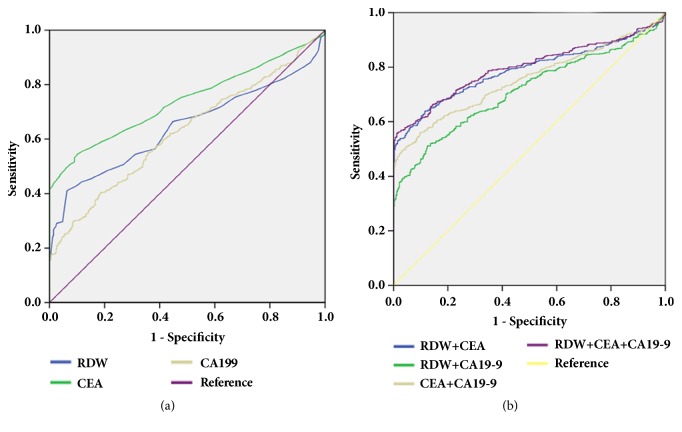
ROC curves of single RDW, CEA, CA 19-9, and the combination in distinguishing CRC patients from healthy participants. (a) ROC curves of single RDW, CEA, and CA19-9 in distinguishing CRC patients from healthy participants. (b) ROC curves of CEA + RDW, CA19-9 + RDW, CEA + CA19-9, and RDW +CEA + CA19-9 in distinguishing CRC patients from healthy participants.

First, we evaluated the value of RDW as a biomarker for the differential diagnosis of CRC patients and healthy participants. As shown in Figure 2 and Table 1, the area under the ROC curve (AUC) for RDW, CEA, and CA19-9 as parameters in the diagnosis of CRC was 0.643, 0.742, and 0.629, respectively. At the cut-off value of 13.95 for RDW, we distinguished CRC patients from healthy participants with a sensitivity of 41% and a specificity of 94 % (Table 1). Importantly, the diagnostic performance of CEA and CA19-9 can be improved when RDW, CEA, and CA19-9 were combined for detection (Table 1). As shown in Figure 2 and Table 1, the AUC for RDW+CEA is 0.789, which was significantly higher than that for RDW+CA19-9 and CEA+CA19-9. Also, the sensitivity of RDW+CEA was significantly higher than that of RDW+CA19-9 and CEA+CA19-9. When three markers are combined, the AUC, the sensitivity, and the specificity of RDW+ CEA+CA19-9 were 0.799, 56%, and 99%, respectively.

**Figure 3:**
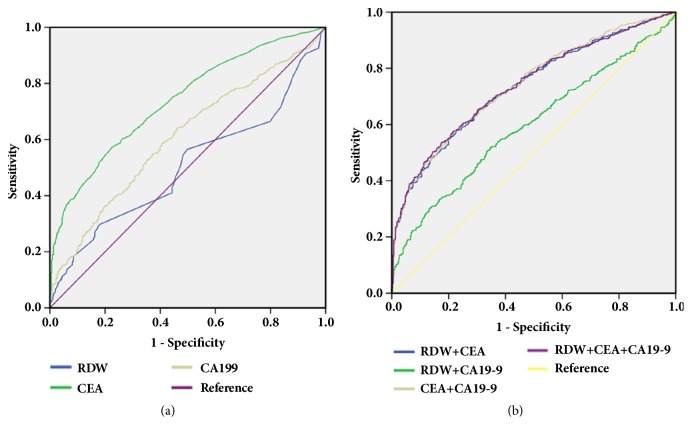
ROC curves of single RDW, CEA, CA19-9, and the combination in distinguishing CRC patients from CA patients. (a) ROC curves of single RDW, CEA, and CA 19-9 in distinguishing CRC patients from CA patients. (b) ROC curves of CEA + RDW, CA19-9 + RDW, CEA + CA19-9, and RDW +CEA + CA19-9 in distinguishing CRC patients from CA patients.

Next, we also performed a ROC analysis to assess the role of RDW in the differential diagnosis between CRC patients and CA patients. CA is a benign glandular tumor of the colon and the rectum and is a precursor lesion of CRC. As shown in Figure 3 and Table 2, the AUC for RDW, CEA, and CA19-9 was 0.502, 0.741, and 0.613, respectively, indicating that RDW has a poor diagnostic performance in the differential diagnosis between CRC patients and CA patients. In addition, RDW did not significantly improve the diagnostic performance of CEA and CA19-9 when RDW, CEA, and CA19-9 were combined for detection (Figure 3 and Table 2).

Taken together, these results suggested that combined detection of RDW, CEA, and CA19-9 provides better diagnostic performance in discriminating CRC patients from healthy participants, with higher sensitivity and specificity. However, RDW had little diagnostic value in the differential diagnosis between CRC patients and CA patients.

### 3.3. Association between RDW Levels and Clinicopathological Characteristics in CRC Patients

As Table 3 shows, RDW levels were significantly associated with TNM stage, pT stages, pM stages, tumor size, and CEA levels. However, no significant association was found between RDW levels and location, age, gender, pN stages, and CA19-9 levels.

## 4. Discussion

In the present study, we found that the level of RDW in blood samples from patients with CRC was significantly higher than those in CA patients and healthy controls. RDW had little diagnostic value in the differential diagnosis between CRC patients and CA patients, but RDW improved the diagnostic performance of CEA and CA19-9 in discriminating CRC patients from healthy participants, with higher sensitivity and specificity. Moreover, the level of RDW was significantly associated with tumor stage, pT stages, pM stages, and tumor size. These findings suggested that RDW has a potential function as a biomarker for the diagnosis and prognosis of CRC.

RDW is one of routine laboratory parameters reported in the complete blood count test. Results from recent studies revealed that RDW might not only be a prognostic marker [4, 6–13], but also be considered as a potential diagnostic marker in various cancer types [14, 15]. Nevertheless, studies about the association between RDW and CRC are limited [15–19], and the value of RDW in CRC is not fully understood. Thus, it is necessary to further investigate the value of RDW in the diagnosis and prognosis of CRC by the larger sample sizes.

In this study, we measured RDW levels in 783 patients with CRC and found that RDW was elevated in patients with CRC, consistent with previous report [18]. ROC analysis suggested that RDW could be a useful marker with a 13.95 cut-off value, 41% sensitivity, and 94% specificity in discriminating CRC patients from healthy participants. CRC is a heterogeneous disease, and it is unlikely that any single marker will have a sufficiently high sensitivity. CEA and CA19-9 have been widely used as tumor markers for the diagnosis and prognosis of CRC. However, the low sensitivity of CEA and CA19-9 limits their clinical use in CRC screening. Our finding showed that combined detection of RDW, CEA, and CA19-9 improved the diagnostic performance of CEA and CA19-9 in discriminating CRC patients from healthy participants. Colorectal adenomas (CA) are precursors of most CRC, and it is difficult to make a differential diagnosis between CRC patients and CA patients. Disappointingly, we found that RDW had little diagnostic value in the differential diagnosis between CRC patients and CA patients; thus further studies are needed to identify a simple, inexpensive, and readily available biomarker for the differential diagnosis of CRC patients and CA patients in the future.

TNM stage, which is based on the extent of the tumor (T), the extent of spread to the lymph nodes (N), and the presence of metastasis (M), is the most predictable prognostic factor for CRC. Our results found that the level of RDW was associated with TNM stage and tumor metastasis; these findings are consistent with the report by Yang et al. [18]. Importantly, we also found that the level of RDW was associated with tumor size. Considering the important value of TNM stage, tumor metastasis, and tumor size in predicting clinical outcome of CRC, our finding suggests that RDW is a potential prognostic biomarker for CRC.

The underlying mechanism that could explain our finding has not been elucidated, but several potential mechanisms may be suggested from previous reports. First, earlier reports have revealed that tumor can lead to the inflammation, and inflammation has a positive correlation with RDW [20–22]. Second, tumor growth can lead to malnutrition, which causes changes in erythropoiesis. Lastly, it is well known that patients with CRC have a tendency for hemorrhage, which reduces iron storages. These reasons all lead to the variation of red blood cell size and the increase of RDW.

Some limitations of this study need to be addressed. First, the short follow-up duration of the study with single-centered retrospective design might raise bias towards sample selection and analysis. Second, we failed to collect data on overall survival; thus we do not evaluate the relationship of RDW with overall survival in CRC patients. Hence, further prospective multicenter studies are needed to validate the clinical significance of RDW in patients with CRC.

## 5. Conclusions

Our study found that the level of RDW was increased in patients with CRC in relatively large sample sizes. Increased RDW not only improved the diagnostic performance of CEA and CA19-9, but also has potential roles as a biomarker for the prognosis of CRC. Considering that RDW is a noninvasive test, and its detection method is economical, fast, and convenient, RDW could be used as an auxiliary biomarker for diagnosis and prognosis of CRC.

## Figures and Tables

**Table 1:** The values of RDW, CEA, and CA19-9 alone and combined biomarkers for distinguishing CRC patients from healthy participants.

Variables	AUC	Cut-off	Sensitivity	Specificity	95% confidence interval	
					Upper limit	Lower limit
RDW	0.643	13.95	41%	94%	0.610	0.673
CEA	0.742	5.00	41%	100%	0.715	0.771
CA19-9	0.629	37.00	17%	100%	0.596	0.662
RDW+CEA	0.789		64%	88%	0.763	0.815
RDW+CA19-9	0.715		51%	88%	0.685	0.744
CEA+CA19-9	0.758		50%	97%	0.731	0.785
RDW+CEA+CA19-9	0.799		56%	99%	0.774	0.823

**Table 2:** The values of RDW, CEA, and CA19-9 alone and combined biomarkers for differential diagnosis of CRC patients and CA patients.

Variables	AUC	Cut-off	Sensitivity	Specificity	95% confidence interval	
					Upper limit	Lower limit
RDW	0.502	14.05	29%	82%	0.469	0.534
CEA	0.741	5.00	41%	90%	0.714	0.768
CA19-9	0.613	37.00	17%	94%	0.581	0.644
RDW+CEA	0.738		57%	78%	0.711	0.765
RDW+CA19-9	0.604		53%	66%	0.572	0.634
CEA+CA19-9	0.744		57%	79%	0.717	0.771
RDW+CEA+CA19-9	0.741		51%	84%	0.714	0.769

**Table 3:** Relationship between RDW and pathological characteristics in CRC patients.

Variables	Total	RDW ⩽ 13.95 %	RDW > 13.95 %	*p*
		n (%)	n (%)	
Sample size	783	465 (59.4)	318 (40.6)	
Gender				0.922
Male	467	278 (59.5)	189 (40.5)	
Female	316	187 (59.1)	129 (40.9)	
Age				0.165
<60	331	206 (62.2)	125 (37.8)	
*⩾*60	452	259 (57.3)	193 (42.7)	
Location				0.097
Colon	430	244 (56.7)	186 (43.3)	
Rectum	353	221 (62.6)	132 (37.4)	
TNM stage				**0.011**
I	136	96 (70.6)	40 (29.4)	
II	247	143 (57.9)	104 (42.1)	
III	322	188 (58.4)	134 (41.6)	
IV	78	38 (48.7)	40 (51.3)	
pT stage				**0.002**
T1	51	40 (78.4)	11 (21.6)	
T2	120	82 (68.3)	38 (31.7)	
T3	481	273 (56.8)	208 (43.2)	
T4	131	70 (53.4)	61 (46.6)	
pN stage				0.248
N0	393	244 (62.1)	149 (37.9)	
N1	240	133 (55.4)	107 (44.6)	
N2	150	88 (58.7)	62 (41.3)	
pM stage				**0.043**
M0	705	427 (60.6)	278 (39.4)	
M1	78	38 (48.7)	40 (51.3)	
Tumor size (cm)				**0.007**
<5	368	237 (64.4)	137 (35.6)	
*⩾*5	415	228 (54.9)	187 (45.1)	
CEA				**0.001**
<5	455	293 (64.4)	162 (35.6)	
*⩾*5	328	172(52.4)	156(47.6)	
CA19-9				0.366
<37	654	393 (60.1)	261 (39.9)	
*⩾*37	129	72 (55.8)	57 (44.2)	

Bold indicates statistical significance.

## Data Availability

The data used to support the findings of this study are included within the article.

## References

[1] Chen W., Zheng R., Baade P. D. (2016). Cancer statistics in China, 2015. *CA: A Cancer Journal for Clinicians*.

[2] Dekker E., Rex D. K. (2018). Advances in CRC prevention: screening and surveillance. *Gastroenterology*.

[3] Thomas D. S., Fourkala E.-O., Apostolidou S. (2015). Evaluation of serum CEA, CYFRA21-1 and CA125 for the early detection of colorectal cancer using longitudinal preclinical samples. *British Journal of Cancer*.

[4] Montagnana M., Danese E. (2016). Red cell distribution width and cancer. *Annals of Translational Medicine*.

[5] Yeşil A., Şenateş E., Bayoğlu İ. V., Erdem E. D., Demirtunç R., Övünç A. O. K. (2011). Red cell distribution width: a novel marker of activity in inflammatory bowel disease. *Gut and Liver*.

[6] Zyczkowski M., Rajwa P., Gabrys E., Jakubowska K., Jantos E., Paradysz A. (2017). The relationship between red cell distribution width and cancer-specific survival in patients with renal cell carcinoma treated with partial and radical nephrectomy. *Clinical Genitourinary Cancer*.

[7] Wei T.-T., Wang L.-L., Yin J.-R. (2017). Relationship between red blood cell distribution width, bilirubin, and clinical characteristics of patients with gastric cancer. *International Journal of Laboratory Hematology*.

[8] Ichinose J., Murakawa T., Kawashima M. (2016). Prognostic significance of red cell distribution width in elderly patients undergoing resection for non-small cell lung cancer. *Journal of Thoracic Disease*.

[9] Kos M., Hocazade C., Kos F. T. (2016). Evaluation of the effects of red blood cell distribution width on survival in lung cancer patients. *Wspolczesna Onkologia*.

[10] Qin Y., Wang P., Huang Z. (2017). The value of red cell distribution width in patients with ovarian cancer. *Medicine*.

[11] Chen G.-P., Huang Y., Yang X., Feng J.-F. (2015). A nomogram to predict prognostic value of red cell distribution width in patients with esophageal cancer. *Mediators of Inflammation*.

[12] Kemal Y., Demirag G., Baş B., Önem S., Teker F., Yücel I. (2015). The value of red blood cell distribution width in endometrial cancer. *Clinical Chemistry and Laboratory Medicine*.

[13] Huang D., Ma R., Xiang Y. (2016). Utility of red cell distribution width as a prognostic factor in young breast cancer patients. *Medicine*.

[14] Beyazit Y., Kekilli M., Ibis M. (2012). Can red cell distribution width help to discriminate benign from malignant biliary obstruction? A retrospective single center analysis. *Hepato-Gastroenterology*.

[15] Spell D. W., Jones D. V., Harper W. F., Bessman J. D. (2004). The value of a complete blood count in predicting cancer of the colon. *Cancer Epidemiology*.

[16] Ay S., Eryilmaz M. A., Aksoy N., Okus A., Unlu Y., Sevinc B. (2015). Is early detection of colon cancer possible with red blood cell distribution width?. *Asian Pacific Journal of Cancer Prevention*.

[17] Kust D., Lucijanic M., Urch K. (2017). Clinical and prognostic significance of anisocytosis measured as a red cell distribution width in patients with colorectal cancer. *QJM: An International Journal of Medicine*.

[18] Yang D., Quan W., Wu J. (2018). The value of red blood cell distribution width in diagnosis of patients with colorectal cancer. *Clinica Chimica Acta*.

[19] Riedl J., Posch F., Königsbrügge O. (2014). Red cell distribution width and other red blood cell parameters in patients with cancer: Association with risk of venous thromboembolism and mortality. *PLoS ONE*.

[20] Dogan M., Kucuk U., Uz O. (2015). Red blood cell distribution width is worthwhile when interpreted with other inflammatory markers. *Journal of Geriatric Cardiology*.

[21] Ephrem G. (2013). Red blood cell distribution width should indeed be assessed with other inflammatory markers in daily clinical practice. *Cardiology*.

[22] de Gonzalo-Calvo D., de Luxán-Delgado B., Rodríguez-González S. (2012). Interleukin 6, soluble tumor necrosis factor receptor I and red blood cell distribution width as biological markers of functional dependence in an elderly population: A translational approach. *Cytokine*.

